# In silico designing and expression of novel recombinant construct containing the variable part of CD44 extracellular domain for prediagnostic breast cancer

**DOI:** 10.1002/cnr2.1745

**Published:** 2022-10-26

**Authors:** Elaheh Gheybi, Ahmad Asoodeh, Jafar Amani

**Affiliations:** ^1^ Department of Chemistry, Faculty of Science Ferdowsi University of Mashhad Mashhad Iran; ^2^ Applied Microbiology Research Center, Systems Biology and Poisonings Institute Baqiyatallah University of Medical Sciences Tehran Iran

**Keywords:** breast cancer, CD44v, recombinant antigen, stem cells

## Abstract

**Background:**

CD44, as a tumor‐associated marker, can be used to detect stem cells in breast cancer. While CD44 is expressed in normal epithelial cells, carcinoma cells overexpress CD44.

**Aims:**

In the current study, we designed a recombinant protein that included the variable component of the CD44 (CD44v) extracellular domain to apply in clinical diagnosis of breast cancer.

**Methods:**

A total of 100 CD44v amino‐acid residues were determined, and the structure was examined using bioinformatics tools. The construct was inserted into the PET28a vector and transformed in *E. coli* BL21(DE3). A nearly 12 kDa fusion protein was obtained by Ni‐NTA affinity metal chromatography. Recombinant CD44v was examined by Western blotting, ELISA, and immunohistochemistry (IHC) assays.

**Results:**

The findings revealed that the structure of rCD44v was stable, and its antigenic domain was exposed. The recombinant CD44v was confirmed by western blotting, and the presence of antibodies against recombinant CD44v protein in the patient's serum was detected by the ELISA. Our data demonstrated a link between CD44v serum levels and the prevalence of breast cancer.

**Conclusion:**

Assessments of antiCD44v antibodies with rCD44v could be a useful tool for identifying breast cancer in its early stages, which can lead to better outcomes.

## 1 INTRODUCTION

Breast cancer (BC), as one of the most frequent cancers in women, is rapidly increasing, particularly in low‐ and middle‐income nations.^1^, ^2^ The cluster of differentiation 44 or CD44, as a tumor‐associated marker, can be used to detect breast cancer stem cells (BCSCs). Normal epithelial cells express CD44, while carcinoma epithelial cells overexpress it. Different CSC properties such as self‐renewability, tumor initiation, metastasis, and chemoradioresistance are regulated by CD44, primarily CD44 variable isoforms (CD44v). As cancer progresses, the total amount spent on therapeutic drugs rises, especially in low‐ and middle‐income countries. Therefore, early intervention is advantageous because it leads to a more successful cure at a lower cost.^3^

Although several available therapies are used to treat BC, some tissue stem cells or their early progenitors are thought to be the source of cancer establishment, leading to cancer reappearance and metastasis years after cure. Self‐renewing cells are a tiny minority of tumor cells that may split into multiple cell types, according to recent research.^4^, ^5^ Cancer stem cells can start a tumor and cause the disease to progress in a limited number of breast cancer populations. In addition, resistance to chemotherapy and radiation contributes to treatment failure and disease relapse; hence, identifying and monitoring BCSCs plays a critical role in prognosis and treatment resistance, potentially opening up new therapeutic options.^1^, ^6^

Many efforts are currently being undertaken to improve advanced diagnostic and therapeutic procedures as well as develop new super tools, such as discovering markers for BCSCs. Important surface indicators in cancer stem cells have recently been found in several investigations.^1^ BCSCs in breast cancer accounts for roughly 2% of all tumor tissue. As a result, getting these cells out of tumor tissue is tough. Many researchers have been hunting for surface breast cancer antigens by identifying the subgroup of BC cells that express CD44^+^/CD24^−^ as a distinctive hallmark of BCSCs.^1^, ^7^ Furthermore, the CD44 CSC surface marker interacts positively with initiator cells.^8^ As a result, the introduction of a complete diagnostic probe for CD44 can lead to early diagnosis and improved treatments.^5^

Antibodies or autoantibodies that can react with tumor cells, tissue, and isolated proteins are generated concurrently with the development of tumor‐dependent antigens, while they are scarcely visible at the early stage. Antibodies, on the other hand, are physiologically increased and quantifiable in the early stages of breast cancer, giving them a viable tool for identifying cancerous tissue from healthy tissue.^9^, ^10^

CD44, which is recognized either as a surface marker of cancer‐initiating cells (CICs) or cancer stem cell antigen, was utilized as a key marker for identifying BCSCs, linking with tumor aggressiveness, metastasis, and recurrence.^5^, ^11^, ^12^ CD44 is a non‐kinase transmembrane glycoprotein having a cytoplasmic carboxyl‐terminal tail and extracellular and transmembrane domains.

The CD44 gene has 19 and 20 exons in humans and mice, respectively; the exon number six and the variant number one have no similarity in humans, while the first and last five exons are fixed in all isoforms and encode the shortest isoform of CD44, known as standard CD44 (CD44s). Furthermore, central exons can be alternatively cleaved and constructed with the 10 exons found in the conventional CD44 isoform, known as “variables,” associated with CD44 variant (CD44v) isoforms, which include the middle nine exons. To make a single variant exon, CD44v isoforms can be coupled with other variant exons that code for extracellular domain peptides.^13^, ^14^

Different CD44v isoforms affect the structure of cell surface as well as the receptors for cytokines and growth factors. CD44's biological activity is influenced by these distinct binding motifs. Hence, cancer cells frequently express a large number of CD44 variations, particularly in the late stages of progression.^11^, ^13^, ^14^ Cell–cell and cell‐substrate interactions, such as proliferation, differentiation, cell migration, cell adhesion, signaling, and survival, are all aided by CD44. Meanwhile, proteolytic enzymes may release this molecule as a cell surface glycoprotein into circulation. As a result, the amount of soluble CD44 proteins in cancer patients' serum correlates with tumor growth and metastasis in various carcinomas.^14^, ^15^, ^16^

CD44 has a high level of expression on bulk tumor cells and CICs, while it has a low level of expression in healthy tissue and differentiated breast tissue, according to the findings.

Up‐regulation of CD44, according to immunohistological research, influences the pace of cell invasion, tumor growth, and metastasis in a variety of malignancies, including breast cancer. Furthermore, patients with CD44 overexpression have had unfavorable outcomes.

Breast cancer treatment frequently has favorable results when compared to other types of early‐stage cancer treatment, making this initial identification critical, and CD44 is the diagnostic target in the early stages of the disease.^1^, ^5^ We assumed that BCSC biomarkers are visible in patients' serums because the most extensively utilized biomarkers, HER2 and MUC1, are employed in breast cancer. Because of the heterogeneity created by the complexity of expression CD44v isoforms, it is critical to screen breast cancers and employ these isoforms as diagnostic biomarkers. CD44v was employed as a BCSC marker in this study to look at the link between serum levels and pathological variables. Our objective was to create a recombinant antigen containing a short common section of variable area exons the extracellular domain (rCD44v) that may be utilized as a coating antigen in immunoassay methods to detect anti‐CD44v antibodies. Also, in this study, we showed that polyclonal antibodies obtained from mice are able to detect CD44v antigen in the serum of breast cancer patients by ELISA method, and we further determined that above antibodies have demonstrated this property in patient's tissue by immunohistochemistry.

## 2 MATERIALS AND METHODS

### 2.1 Design of antigen structure

The CD44 variants sequences were retrieved in FASTA format from UniProt protein databases (P16070) and aligned using Clustal W software to find the conserved areas of human CD44. Nearly 100 amino acids were taken from various areas of the CD44 extracellular domain regions to make a single synthetic protein. The modified protein's physicochemical characteristics were investigated using the ExPASy server's ProtParam command. The special specialized server GOR‐IV and I‐TASSER predicted the secondary and tertiary structures of proteins, respectively.

Likewise, the 3D‐protein models were examined by comparative modeling like Mod‐Base and Loop server. To predict continuous B‐cell epitopes, the amino acid sequences were examined using the Bcepred, and ABCpred programs. Simultaneously, to predict discontinuous B‐cell epitopes, Discotope 1.2 and SEPPA were applied. To predict antigenic sequence binding MHC Class I and T‐cell epitopes, CTLPRED was used.

HLApred, MHCpred, and ProPred were used to predict peptides from the recombinant protein binding MHC Class ll. The amino acid sequence of hCD44 was reversely translated into nucleotide sequence, and codons were optimized by the General biosystem service. Ultimately, the secondary structure of mRNA was predicted and analyzed by the mfold program.^17^, ^18^, ^19^

### 2.2 Cases and controls

A total of 67 tissue and serum samples were taken, 30 healthy women with an average age of 43 and an age range of 26 to 60 years, four patients with benign breast tumors with an average age of 37, and an age range of 26 to 49 years, and 33 breast cancer patients with an average age of 49 and an age range of 28 to 71 years, before and after treatment including surgery, post‐operative radiotherapy, and, or chemotherapy from Imam Khomeini Hospital (Tehran, Iran). We screened 25 samples of positive antigens and 12 samples of negative antigens for CD44 by immunohistochemistry using mouse monoclonal Ab anti‐human CD44 (DAKO, Catalogue # M 7082).

The BC patients encompassed 4 benign tumor persons with hyperplasia and fibroadenoma and 33 persons with adenocarcinoma. Meanwhile, the healthy samples included 30 nonpregnant and non‐lactating samples (Table 1).

**TABLE 1:** Patients' properties

Patients' properties	
Healthy women	30
Total patients	37
Age	28–71 years
Stages	III and IV
Estrogen receptor	
Positive	17
Negative	20
Progesterone receptor	
Positive	18
Negative	19
HER2	
Positive	9
Negative	28
CA15‐3	
Positive	22
Negative	15
CD44	
Positive	25
Negative	12

### 2.3 Expression and purification of CD44v

To create the pET28‐CD44v construct, the 107‐amino‐acid designed sequence was synthesized and cloned into the pET28a (General biosystem service). GeNetBio kit was used to extract plasmids (Catalogue # K‐1000). A PCR reaction with T7 universal primers was carried out to validate the pET28a‐CD44 construct. Furthermore, the sequence of construct was presented and confirmed by General biosystem service. The construct was used to convert *E. coli* BL21 (DE3) competent cells using a thermal shock technique.^20^ Four milliliter of pET28a‐CD44 harboring *E. coli* BL21 (DE3) was inoculated into 200 ml LB broth encompassing 25 μg/ml kanamycin (Sigma‐Aldrich, Catalogue # K1637) and incubated at 170 rpm and 37°C for 2 h to reach OD600 to 0.7–0.9, then 1 mM IPTG (Sinaclon, Catalogue # CL5812) was added. The six His‐tagged fusion protein of CD44 (rCD44v) was subjected to Ni‐NTA column chromatography (Qiagen, Catalogue # 30210) under native conditions.^21^ Different concentrations of imidazole(Merck, Catalogue # 104716) followed by a pH gradient procedure were used to elute the rCD44v antigen. Non‐induced recombinant cells were considered as the negative control; 15% SDS‐PAGE was used to check the purity, and protein content was estimated by the Bradford method.^22^

### 2.4 Recombinant protein confirmation by western blotting

The purified antigen was electrophoresed on gel 15% SDS‐PAGE and transferred to a polyvinylidene difluoride (PVDF) membrane using transfer buffer. After running, the membrane was blocked by 5% BSA (Sigma, Catalogue # 7030) at 37°C for 1 h. Thereupon, the PVDF membrane was washed three times with 0.05% tween 20 in PBS buffer (PBST). The recombinant CD44v was incubated with a mouse antibody against His‐tag conjugated with HRP (1/2000, Roche, Catalogue # 11965085001) in PBST buffer and was shaken at 37°C for 1 h and then washed with PBST buffer several times. In the next step, the detection was performed using an HRP staining solution (DAB) (Diagnostic Biosystems, Catalogue # K047). The chromogenic process was finally stopped by washing twice with pure water.^23^

### 2.5 Preparation of anti‐CD44v serum

Twenty micrograms of recombinant CD44v protein was injected subcutaneously into the back of necks of 5‐week‐old female BALB/c mice with complete Freund's adjuvant (Sigma, Catalogue # F5881).

On days 14 and 28, an incomplete Freund's adjuvant (Sigma, Catalogue # F5506) was utilized as a booster dosage in future injections. Furthermore, as a negative control, full and incomplete Freund's adjuvant was administered in the same way as previously. The indirect ELISA technique was used to detect antibodies against the rCD44v antigen, and then the mouse serum was collected and kept at −70°C for future usage.^24^

### 2.6 Immunohistochemical analysis

The prepared mouse polyclonal antibody (pAb) against rCD44v and monoclonal antibody (mAb) against hCD44 (DAKO, Catalogue # M 7082) were used to stain the breast cancer tissue according to standard Immunohistochemical (IHC) protocol. Slides were incubated with mouse pAb against rCD44v, mAb against human CD44 diluted with TBST (Tris‐borate saline‐ tween 20) in a 1:100 ratio. After washing the slides by TBST for 5 min and they were incubated with HRP conjugated anti‐mouse antibody (Diagnostic Biosystems, Catalogue # PVP100D) for 30 min. The slides were incubated with 3, 39‐diaminobenzidine tetrahydrochloride (DAB) (Diagnostic Biosystems, Catalogue # PVP100D) and instantly washed by tap water after color development. The slides that were not incubated with anti‐rCD44v antibodies were considered as a second control. All slides were immersed in hematoxylin for 3 min and then rinsed with running water. After that, dewatering, clarification, and gluing of the slide were performed, respectively, and ultimately the slides were evaluated under a light microscopy (Olympus BX51).^25^, ^26^

### 2.7 Determination of serum IgG antibody against rCD44v

Specific antibody responses in mouse samples and patients' sera were measured using ELISA, in triplicate. To do so, 5 μg of purified rCD44v was diluted in coating buffer (Na_2_CO_3_, NaHCO_3_, pH 9.6) and held at 4°C for 16 h on 96‐well plates (Nunc). After that, the plates were washed three times with PBST and blocked for 1 h at 37°C with 5% BSA (Sigma, Catalogue # P 7030). The mouse control and test serum samples, mAb against CD44 (DAKO, Catalogue # M 7082) diluted (1:50) by PBST, and 37 patients' sera samples diluted (1:25) by PBST, were poured into wells and incubated at 37°C for 1 h. In the following, they were washed by PBST several times and conjugated HRP with goat anti‐mouse IgG and anti‐human IgG secondary Abs (1/2000 in PBST) (Razi BioTech, Catalogue # AP8036 and Catalogue # AP7071, Kermanshah, Iran) was added to the wells at 37°C for 1 h and washed three times by PBST. Afterward, 100 μl of substrate solution containing 0.06% (wt/vol) O‐phenylenediaminedihydrochloride (OPD) (Sigma, Catalogue # P1526) and 0.06% (vol/vol) H_2_O_2_ (DNAbiotec, Catalogue # DB9651, Pretoria, South Africa) were added to the plates and placed at ambient temperature for 15 min in the dark. Ultimately, the reaction was terminated with 100 μl of 2 M H_2_SO_4_, and the OD at the wavelength of 492 nm was recorded by an ELISA reader (BioTek, ELX800).^26^, ^27^, ^28^

### 2.8 Quantitative measurement of serum CD44v by ELISA

Microplates coated with 2 μg rCD44v were serially diluted to 1:100 by coating buffer (pH 9.6) and placed at 4°C for 16 h. Next, the microplates were blocked with 5% BSA solution at 37°C for 1 h. After washing by PBST, mice serums containing anti‐rCD44v antibodies were poured into each well at a dilution of 1/100, then incubated at 37°C for 1 h, and washed again.

Next, anti‐mouse IgG conjugated with peroxidase (Razi BioTech, Catalogue # AP8036) was applied to the wells at a dilution of 1/2000 and incubated for 1 h at 37°C, followed by the addition of OPD as a peroxidase substrate, the color was developed, and OD492 was recorded. A standard curve was created to measure the relative values of rCD44v in serum samples, and the optical density of CD44v antigen in patients' serum was measured using the standard curve.

### 2.9 Statistical analysis

The data are obtained from three independent experiments and analyzed using SPSS version 17.0 (SPSS Inc.), as mean ± standard deviation. For multigroup comparisons, the statistical significance was determined using the Two‐way ANOVA test (*p*‐value >.05).

## 3 RESULTS

### 3.1 Structural design and prediction

**FIGURE 1:**
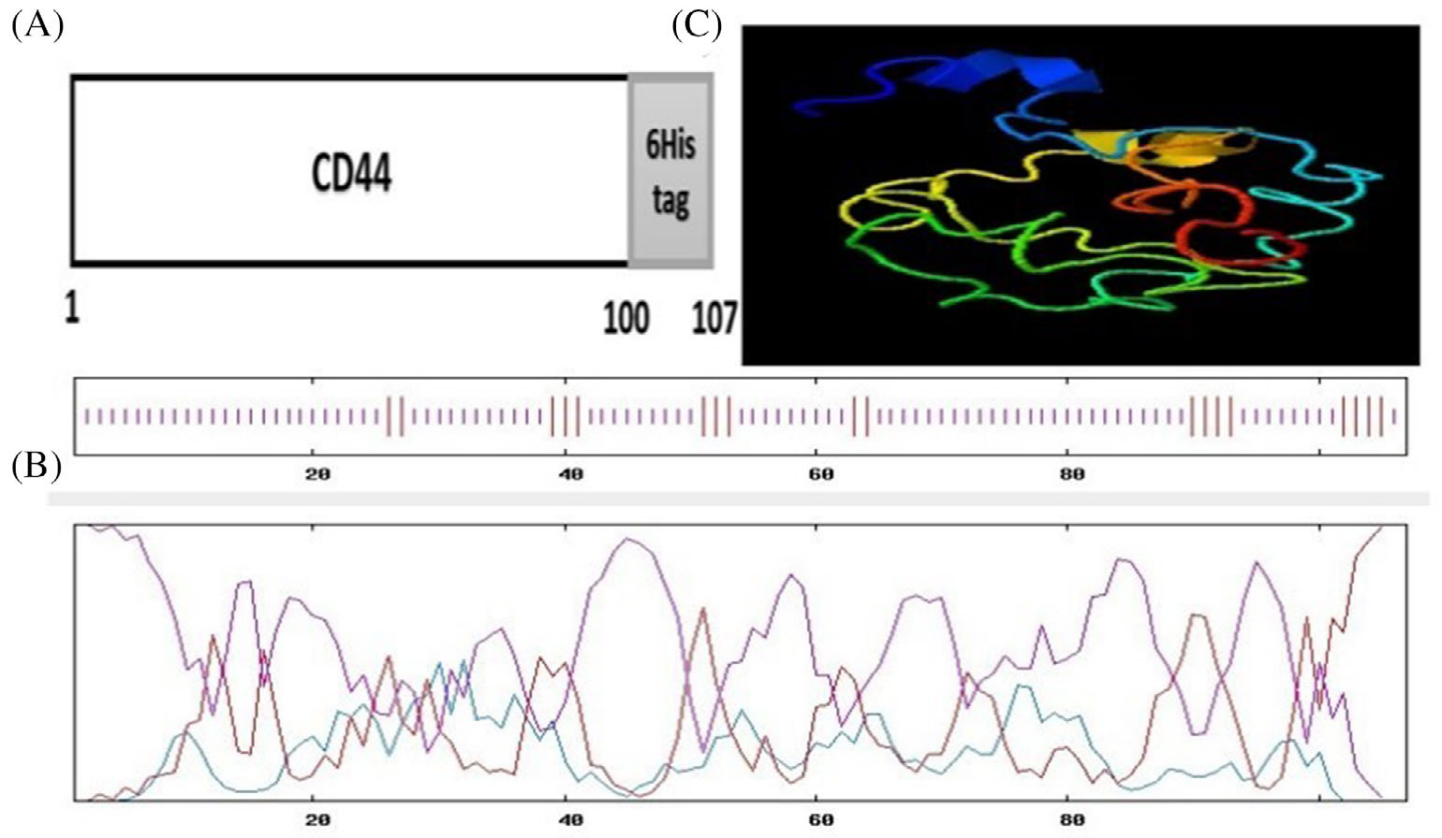
Designing construct. (A) Schematic view of recombinant constructed CD44v protein. (B) Secondary structure analysis of the rCD44v, extended strand (purple): 16.82%, random coil (red): 83.18%. (C) The models are predicted by the I‐TASSER server and visualized by Discovery Studio viewer.

A short common section of variable area exons the extracellular domain of hCD44 containing the N‐terminal 100 amino acid residues (441–540) was chosen for assembly in this investigation. As a fusion tag, six His amino acids were utilized at the C‐terminus (Figure 1A). With the accession number MT396985, the designed sequence was deposited in Genbank. The recombinant protein's physiochemical characteristics comprised a molecular mass of 12 kDa, a pI of 5.65, and aliphatic indexes of 36.45. Figure 1 shows predictions for the second and third recombinant protein shapes. Secondary structure predictions validated the high fraction of random coils (83.18%) and low extended strand (16.82%) (Figure 1B), while tertiary structure predictions revealed the immunogenic region was exposed (Figure 1c) and antigenic residues had VaxiJen cutoff values of >0.5.

### 3.2 Other structural features

Algpred tool and SDAP allergen library were applied to predict the allergenicity of sequences. According to their results, there is no potential for allergenicity of rCD44v antigen.

### 3.3 Prediction of B and T cell epitope

Table 2 summarizes the primary epitope properties, including hydrophilicity, accessibility, stability, antigenicity, polarity, and exposed recombinant protein surfaces. Table 3 also displays the anticipated B and T cell epitopes with the greatest interaction score for both MHC Classes I and II.

**TABLE 2:** Different epitopes predicted in CD44 based on different parameters using the Bcepred database

Prediction parameters	Epitope segments
Hydrophilic	TTPSPEDSSWTD,GRGHQAG, DMDSSHS, QPTANPNTG, EDLDRTG, MTTQQSNSQS,HEGLEEDKDHPTTST,TSSNRNDV
Flexibility	MTTPSPED,LSMTTQQSNSQSFSTSH,PTTSTLTSSNRN
Accessibility	MTTPSPEDSSWTD,RGHQAGRRMDMDS,QPTANPNT,VEDLDRTGP, SMTTQQSNSQSF,HEGLEEDKDHPTTSTLTSSNRNDV
Exposed surface	LEEDKDHPT
Polarity	HPMGRGHQAGRRMDMDS,LVEDLDRTG,TSHEGLEEDKDHPTT
Exposed surface	LEEDKDHPT

**TABLE 3:** Prediction of B and T cells epitopes using ABCpred and CTLpred database

	B cell epitopes prediction		
Rank	Sequence	Score	Position
1	TLQPTANPNT	0.79	41
2	SSHSITLQPT	0.78	36
3	QAGRRMDMDS	0.75	27
4	SSWTDFFNPI	0.72	9
5	LVEDLDRTGP	0.72	52
6	FFNPISHPMG	0.66	14
7	MTTPSPEDSS	0.66	1
8	RTGPLSMTTQ	0.57	58
9	HPMGRGHQAG	0.55	20

	T cell epitopes prediction		
Rank	Sequence	Score	Position
1	EDSSWTDFF	0.99	1–7
2	QPTANPNTG	0.99	2–43
3	GLVEDLDRT	0.98	3–51

### 3.4 Expression, purification of rCD44v protein

**FIGURE 2:**
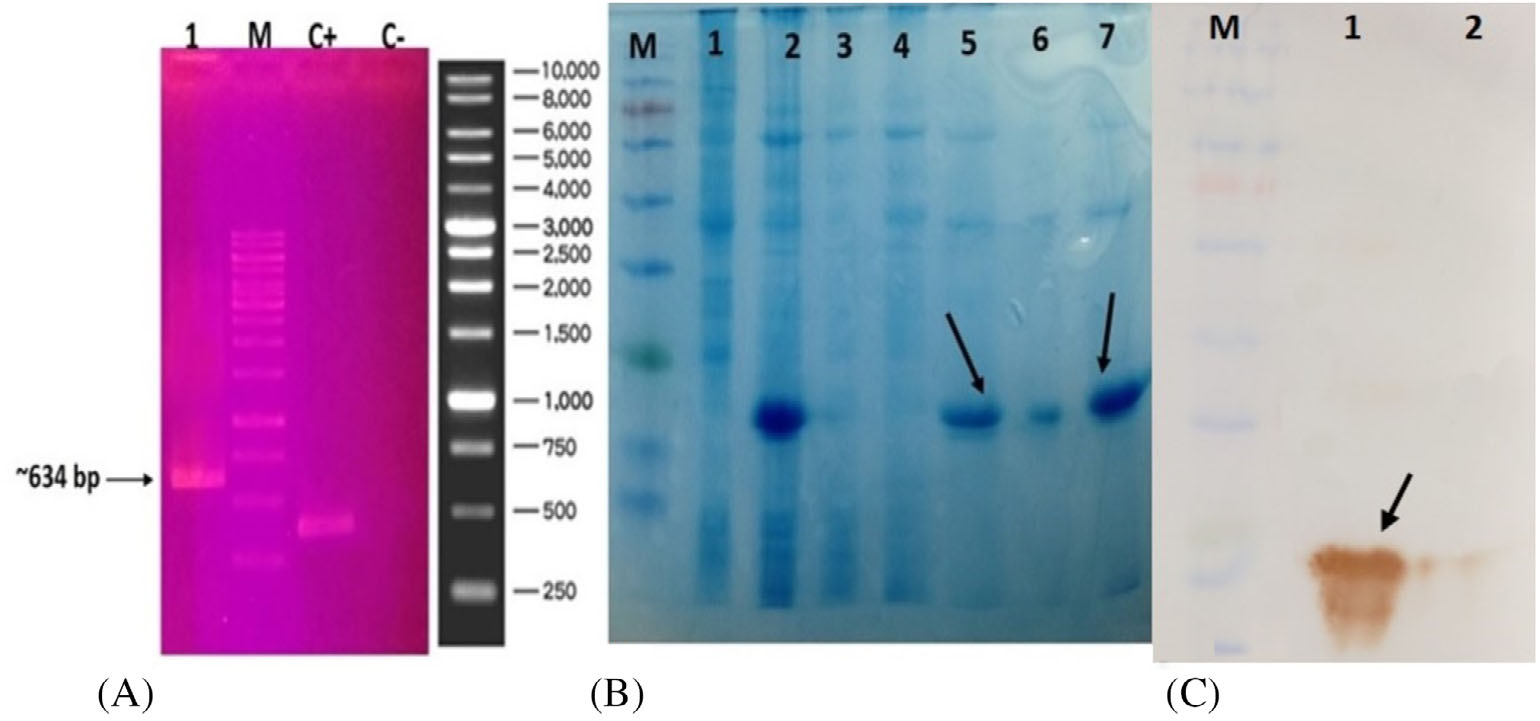
Expression and purification of recombinant CD44v. (A) 1% Agarose gel electrophoresis analysis PCR reaction with promoter and terminator primers T7 universal of the pET28 vector. Lane 1: confirmation construct gene CD44v in the vector, Lane 2: 1 kb ladder. (B) Purification of recombinant CD44v protein on SDS PAGE. Lane M, molecular weight marker. Lane 1, un‐induced *E. coli* as control. Lane 2, before column. Lane 3, flow through. Lane 4, washing with 40 mM imidazole. Lanes 5, 6, elution with 250 mM imidazole, and Lane 7, 20 mM MES buffer. (C) Confirmation of recombinant CD44v protein by western blotting. Lane M, molecular weight marker. Lane 1, recombinant CD44v. Lane 2, un‐induced *E. coli* as control

The PCR reaction was performed with T7 promoter primer and T7 terminator primer in terms of temperature and time in three steps which are: initial denaturation at 95°C for 5 min, (35 cycles) at 94°C 20 s, 54°C 20 s, 72°C 20 s, and final extension at 72°C for 5 min. The PCR products were examined by electrophoresis on agarose gel 1%, and a cloned gene fragment of size 324 bp was observed (Figure 2A). The expressed recombinant CD44v was purified by Ni metal affinity chromatography, and fractions were examined by SDS‐PAGE 15%, and results showed a band of about 17 kD (Figure 2B). The accuracy of recombinant CD44v was verified by reaction with anti‐His tag Abs by western blotting (Figure 2C). In the negative control, however, there was no reactivity (Figure 2C).

### 3.5 Detection of specific mouse Abs against CD44v with ELISA and IHC

**FIGURE 3:**
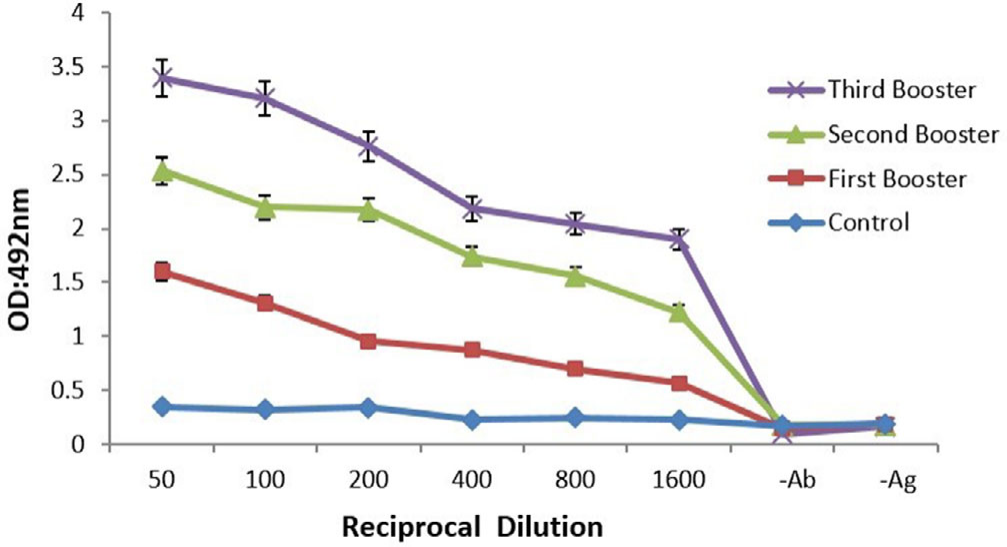
rCD44v‐specific serum IgG following subcutaneous immunization. Mice were injected with recombinant CD44v using complete and incomplete Freund's adjuvants. Immunizations were performed three times within 6 weeks. The sera were collected after immunization and assessed for rCD44v‐specific IgG by the ELISA method. Non‐immunized mice sera were used as control (*p* < .05).

**FIGURE 4:**
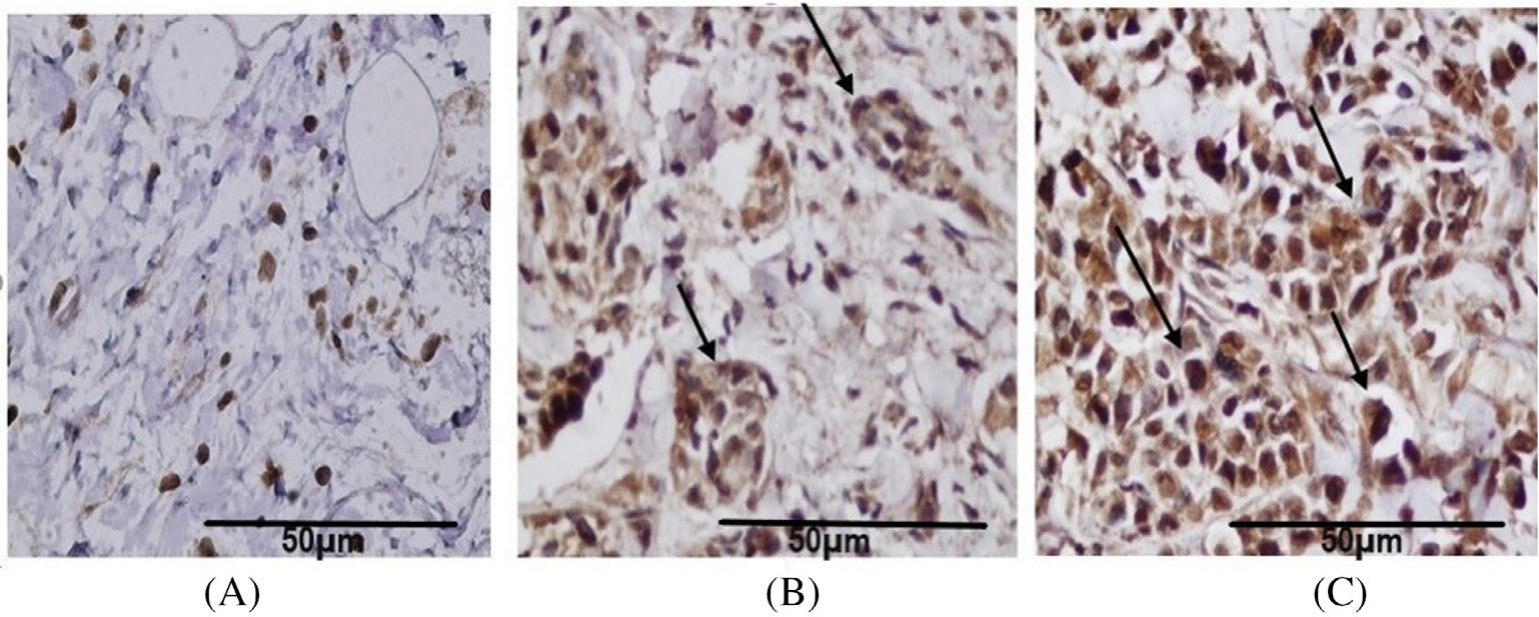
Analysis of mouse anti‐rCD44v antibodies for detecting antigen. (A) Immunohistochemically staining of normal breast, (B) breast cancer sections staining by standard anti‐CD44, (C) breast cancer sections staining by mouse anti‐rCD44v. All Immunostaining images are captured at magnification ×40, scale bar 50 μm

Immunization of mice with pure rCD44v protein resulted in the formation of specific IgG antibodies. Compared with mice control antisera, an anti‐rCD44v IgG antibody titer can be detected even at 1/1600 dilutions (Figure 3). Our findings revealed that rCD44v can be detected by either anti‐CD44 or anti‐His‐tag antibodies. Furthermore, IHC examination exhibited that the mice anti‐CD44 polyclonal Ab, like typical anti‐CD44 monoclonal Ab, can detect CD44 protein expressed in breast tumors (Figure 4).

### 3.6 Detection of CD44v protein in patients' sera

**FIGURE 5:**
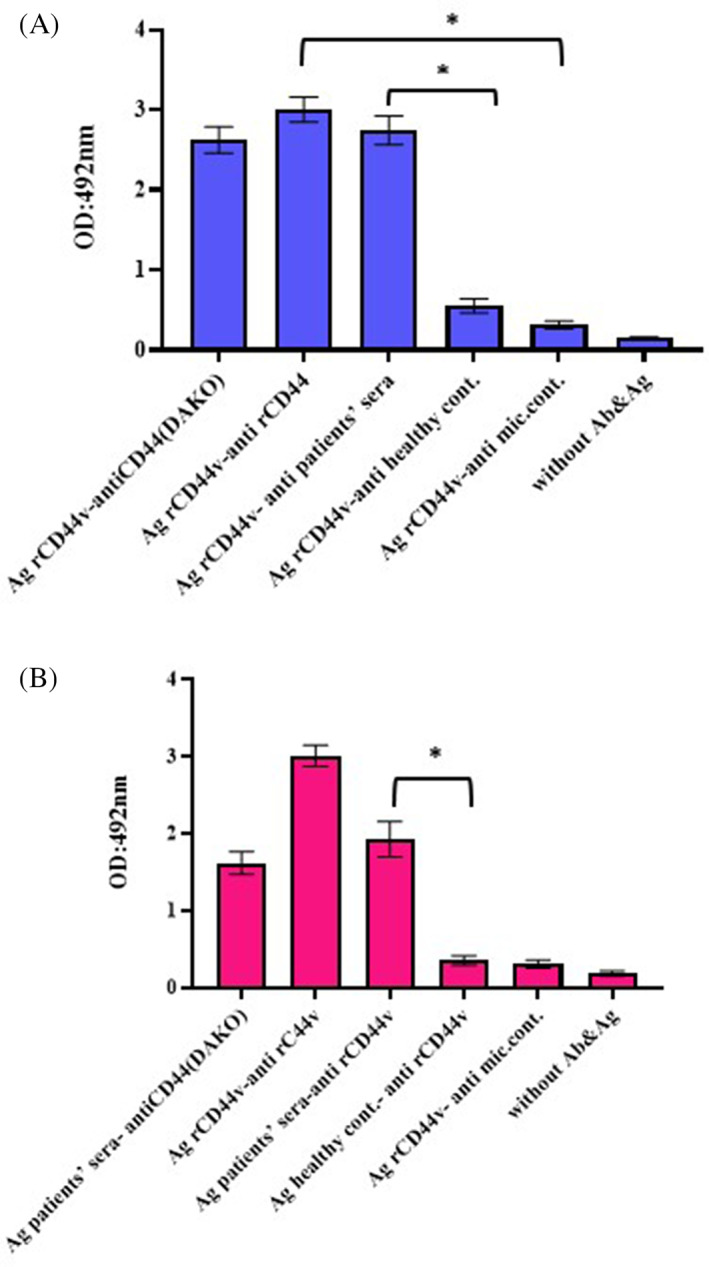
(A) An ELISA test was performed using rCD44v as a coating antigen, with anti‐CD44v IgG in the patients' sera and healthy controls along with the serum of test and control mice, commercial anti‐CD44 as reference (*p* < .05). (B) An ELISA was established using CD44v antigens in the patients' sera and healthy controls along with the serum of test and control mice, as a coating antigen with mouse anti‐rCD44v antibody, commercial anti‐CD44 as reference (*p* < .05). These experiments were run in triplicate

ELISA analysis revealed that rCD44v protein was capable of detecting anti‐CD44v antibodies in patients' serum. This means that natural anti‐CD44v antibodies can recognize epitopes on rCD44v (Figure 5A). In addition, anti‐rCD44v antibody identified CD44v proteins in patients' serum on the plate (Figure 5B and Table 1).

### 3.7 Calculating CD44v antigen in the patients' sera by ELISA

ELISA was used for quantitative measurement of CD44v antigen in serum by comparing their optical densities with a standard curve and calculating relative values. We considered the cut‐off value of 631.3 ng/ml as the positive quotient of CD44v, which is values higher than 631.3 ng/ml are considered positive CD44v. Thus, the sensitivity and specificity of this procedure were determined through SPSS analysis 92% and 98%, respectively.

## 4 DISCUSSION

A biomarker demonstrates physiological changes from a healthy state to illness, pharmacological therapy, toxin presence, and other environmental stressors. Cancer‐associated autoantibodies are regarded as ideal biomarkers, since they reflect the immune system's biosensors and are known to respond to cancer development. Immunoglobulins are highly stable in serum samples and can last for a long period after the antigen has been removed. They have a major advantage over other bodily fluid measures when used.^9^ Like many other tumors, early diagnosis of breast cancer is critical in responding well to the treatment and lowering the mortality rate. CD44, a surface biomarker extracted from CICs, is among the most effective biomarkers for the early identification of breast cancer.^5^ CICs and stem cells have many similarities in terms of niche interaction, epithelial‐mesenchymal transformation (EMT), motility, and apoptosis resistance, all of which rely on CD44.

Cell adhesion, required for the growth of multicellular organisms, stem cell adhesion, embryonic migration, and tissue healing, is depended on activities of CD44 on the surface of CICs. CD44v, unlike CD44s, is only found on a few epithelial cells and in a few kinds of cancer. CD44 (possibly CD44v) has important biological activities for CICs, and there is a link between incremental CD44 isoform regulation and tumor development to the metastatic phase and breast cancer recurrence. In parenthesis, the CD44 isoforms implicated are also listed (CD44v3, CD44v4, CD44v5, CD44v6, CD44v7, CD44v10).^26^

In this study, we cloned and expressed a novel short common sequence of variable extracellular domain human CD44 (variant isoforms 3, 4, 5, 6, 8, 9, and 10) in the pET28a vector. We have developed a reliable ELISA method for detecting serum auto‐antibodies against CD44v, the advantages of our designed molecule are getting a more significant number of epitopes and greater efficiency in coating plates, natural antigenicity without glycosylation, high specificity, and crucial property as a coating antigen to detect anti‐CD44v antibodies in clinical samples.^9^, ^10^

To test the sensitivity and specificity of ELISA, we devised two separate techniques, one for detecting anti‐CD44v antibodies and the other for detecting CD44v antigens in patients' sera. When serum samples were diluted at 1:25, the results indicated that both techniques had identical detection rates. But, immune responses create huge amounts of antibodies with extended half‐lives, allowing them to be tracked with great sensitivity. Antigens found on malignant or premalignant lesions, on the other hand, are often generated on a tiny scale and are not detectable in the blood due to dilution or clearance.^10^, ^29^, ^30^ The rate of anti‐CD44 IgG was greater in breast cancer patients' pre‐and post‐surgery than in healthy persons, according to the comparative diagnostic advantage of the CD44.^9^, ^31^ Senbanjo LT et al. discovered a link between blood soluble CD44 concentrations in patients and tumor load and metastasis in BC patients.^32^ Also, Isamu Okamoto et al. discovered significant quantities of CD44 in normal breast specimens, however considerably lower than in tumor tissues.^30^ Notably, ectodomain cleavage of CD44 is particularly common in tumors, and tumors expressing a CD44 splice variant have an increase in CD44 cleavage; thus, the high prevalence of CD44 cleavage suggests that it plays a key role in tumorigenesis.^12^, ^30^

Although comparable effects have been reported in other cancer patients, it has been revealed that therapy successfully lowered circulating anti‐CD44 antibody levels in breast cancer patients.^9^, ^10^, ^33^ Following the therapy, the circulating anti‐CD44 IgG in breast cancer patients was depleted in proportion to the period before treatment.

During extended illness and latent postoperative micro‐metastasis initial treatment, the consequence of necrosis or metalloproteases, which release membrane‐bound antigen into the blood, resulting in reduced levels of circulating anti‐CD44 antibodies.^2^, ^13^, ^31^

Although there has been a negative association between circulating anti‐CD44 antibodies and antigen in patients with specific malignant tumors, CD44 antigen exhibited a substantial negative link with the matching antibody in breast cancer patients in our current investigation. Meanwhile, there is no correlation between HER2 and CA15‐3 levels and CD44, based on patient characteristics (Table 1) and our findings.^29^, ^30^, ^31^

Our findings suggest that low and high CD44v antigen levels in individual patients may be linked to low and high anti‐CD44v antibody levels, respectively. Synchronized elevation of CD44v antigens and anti‐CD44v antibodies in serum can be expected when breast cancer has progressed to a late stage. As a result, anti‐CD44v antibodies may bind to CD44v antigen in breast cancer patients, implying that recurrence or distant metastasis may play a role in CD44v antigen overexpression.^13^, ^27^, ^29^

To summarize, we used two ELISA techniques to look for benign and malignant breast cancers in blood samples. Recognizing the CD44v antigen, according to our findings, is required in breast cancer, particularly in malignant tumors. In addition, assessing the quantity of anti‐CD44v antibodies in the blood can be a useful tool for identifying breast cancer in its early stages, which leads to better outcomes. To expand the study, further extensive investigation with more instances is required.

## AUTHOR CONTRIBUTIONS

**Elaheh Gheybi:** Conceptualization (equal); data curation (equal); formal analysis (equal); investigation (equal); methodology (equal); writing – original draft (equal); writing – review and editing (equal). **Ahmad Asoodeh:** Investigation (equal); project administration (equal); supervision (equal); writing – review and editing (equal). **Jafar Amani:** Project administration (equal); supervision (equal); writing – review and editing (equal).

## FUNDING INFORMATION

This study was supported by the Research and Technology Council of the Ferdowsi University of Mashhad, Mashhad, Iran (Grant number: 3/48488, 1397/10/11).

## CONFLICT OF INTEREST

The authors have stated explicitly that there are no conflicts of interest in connection with this article.

## ETHICS STATEMENT

The present study was approved by the Ethics Committee of Ferdowsi University of Mashhad (Mashhad, Iran), and conducted according to the 1964 Declaration of Helsinki.

## CONSENT TO PARTICIPATE

Informed consent was obtained from all individual participants included in the study.

## Data Availability

The data that support the findings of this study are available from the corresponding author upon reasonable request.
